# Signal transducer and activator of transcription 3 signaling upregulates fascin via nuclear factor-κB in gastric cancer: Implications in cell invasion and migration

**DOI:** 10.3892/ol.2014.1804

**Published:** 2014-01-15

**Authors:** JUN YAO, CUI-JUAN QIAN, BEI YE, ZHI-QIANG ZHAO, JIE WEI, YONG LIANG, XIN ZHANG

**Affiliations:** 1 Institute of Tumor, School of Medicine, Taizhou University, Taizhou, Zhejiang 318000, P.R. China; 2 Insitute of Gastroenterology, Sir Run Run Shaw Hospital, Zhejiang University, Hangzhou, Zhejiang 310016, P.R. China; 3 Department of Gastroenterology, Taizhou Municipal Hospital, Taizhou, Zhejiang 318000, P.R. China

**Keywords:** gastric cancer, fascin, signal transducer and activator of transcription 3, nuclear factor-κB, Notch, metastasis

## Abstract

Fascin protein plays important roles in tumor metastasis and is prognostically relevant to human gastric cancer (GC). However, its role in the development and progression of GC has not been comprehensively investigated. In the present study, results revealed that upregulation of fascin by interleukin-6 promotes GC cell migration and invasion in a signal transducer and activator of transcription 3 (STAT3)-dependent manner in MKN45 cells. Furthermore, STAT3 directly regulated fascin expression and nuclear factor-κB (NF-κB) bound to the fascin promoter in a STAT3-dependent and Notch-independent manner. Therefore, results demonstrate that STAT3 and NF-κB are required for upregulation of fascin and for cell migration and invasion in MKN45 cells. Effects of the treatments on cell signaling were detected by qPCR, western blot analysis and chromatin immunoprecipitation (ChIP) assay. Cell migration and invasion were analyzed using in vitro scratch wound healing assay, transwell and Matrigel assays, and xenograft model. In addition, the STAT3-NF-κB-fascin signaling axis is identified as a therapeutic target for blocking GC cell invasion and migration.

## Introduction

Although the incidence and mortality of gastric cancer (GC) have markedly decreased worldwide over the last 50 years, it remains the world’s second leading cause of tumor-related mortality (1). Despite advances in diagnosis and treatment, the majority of patients with advanced GC will succumb to the disease, due to local tumor invasion and distant metastasis (2). Tumor metastasis is a multistep process and is regulated by a number of growth factors and cellular signaling pathways (3,4). Fascin is an actin-bundling protein that is important for the maintenance and stability of filamentous actin bundles, and is consequently involved in cell motility (5). Fascin expression is low or absent in the majority of normal adult epithelia and often upregulated in various types of tumor, for example breast, prostate and brain tumors, bladder cancer and esophageal squamous carcinoma (6,7). Tumor cells with high expression of fascin have been found to exhibit increased membrane protrusions and migration ability (6,7), suggesting that fascin is associated with clinical aggressiveness and metastasis. However, the mechanism by which fascin expression is upregulated in tumors is not understood. Although the prognostic relevance of fascin expression has been reported in human GC (8), the present study aimed to investigate the relationship between fascin expression and cell migration and invasion in GC cells. Through this, is was hoped to further elucidate the underlying molecular mechanisms.

Signal transducer and activator of transcription 3 (STAT3) is a well-known transcription factor and regulates a variety of cellular processes, including cell proliferation and survival, oncogenesis and cancer metastasis in GC. Dysregulation of STAT3 signaling is a frequent cause of gastric carcinogenesis (9–11). Interleukin-6 (IL-6), a member of the glycoprotein 130 family of cytokines, can induce activation of STAT3 signaling pathways to affect downstream signaling and cellular events (12,13). Furthermore, study results suggest that IL-6 promotes GC metastasis through activation of STAT3 (9,11) and that inhibition of STAT3 blocks angiogenesis and metastasis of GC (14). However, the mechanism by which IL-6-induced STAT3 activation promotes GC metastasis is not well defined. Additionally, IL-6 promotes cell invasion by upregulating fascin expression in glioblastoma cells (15), suggesting a possible correlation between STAT3 activation and fascin expression. However, whether STAT3 induces fascin expression in GC is currently unknown, and the roles of fascin in the malignant behavior of GC remain unclear.

## Materials and methods

### Cell culture

Five human gastric carcinoma cell lines, MKN45, MKN28, BGC823, AGS and SGC7901, were cultured in RPMI-1640 medium (Invitrogen Life Technologies, Carlsbad, CA, USA) supplemented with 10% fetal bovine saline (FBS). MKN45 cells were subsequently treated with various concentrations of recombinant human IL-6 (R&D Systems, Minneapolis, MN, USA) for the indicated durations in their respective starvation mediums. A human gastric epithelial cell line, GES-1, was cultured in Dulbecco’s modified Eagle medium (Invitrogen Life Technologies) supplemented with 10% FBS.

### Reagents

Janus kinase 2 (JAK2) inhibitor (AG490) and γ-secretase inhibitor (DAPT) were purchased from Calbiochem (La Jolla, CA, USA). Dimethyl sulfoxide (DMSO) and DAPI were purchased from Sigma-Aldrich (St. Louis, MO, USA). Antibodies used for western blotting and chromatin immunoprecipitation (ChIP) assays were anti-phosphotyrosine (p)STAT3 (Cell Signaling Technology, Inc., Beverly, MA, USA), anti-STAT3 (Cell Signaling Technology, Inc.), anti-nuclear factor (NF)-κB p50 (Santa Cruz Biotechnology, Inc., Santa Cruz, CA, USA), anti-fascin (Abcam, Cambridge, UK), anti-hairy and enhancer of split-1 (Hes-1; Abcam), anti-activated-Notch1 (Abcam), anti-activated-Notch2 (Abcam), anti-matrix metalloproteinase (MMP)-2 and anti-MMP-9 (Cell Signaling Technology, Inc.), anti-GAPDH (Abcam), anti-rabbit immunoglobulin (Ig)G and horseradish peroxidase-linked antibody (Cell Signaling Technology, Inc.).

### ChIP assays

ChIP assays were performed using a ChIP assay kit, according to the manufacturer’s instructions (Upstate Biotechnology, Lake Placid, NY, USA) as described previously (16,17). Briefly, cells were treated as indicated and cross-linked with formaldehyde and sonication. Resulting cell lysates (input) were immunoprecipitated with 2.5 μg STAT3 antibody, NF-κB p50 or normal rabbit IgG. The precipitated protein-DNA complexes (IP) were subjected to proteinase treatment. The primers used to confirm the binding of factors to the promoter region of fascin had the following sequence: were 5′-accttgtgggcagcctgt-3′ and 5′-attccctgcagacaccacct-3′.

### Reverse transcription and quantitative (q)PCR

Reverse transcription and qPCR were performed as described previously (16–18). RNA was extracted with TRIzol reagent (Invitrogen Life Technologies) and concentrations of RNA were quantified by NanoDrop 1000 (NanoDrop, Wilmington, DE, USA). Reverse transcription was performed using the Reverse Transcription System (Promega GmbH, Madison, WI, USA) to obtain cDNA, which was subjected to qPCR using SYBR^®^ Premix *Ex Taq*™ (Takara Bio, Inc., Shiga, Japan) on a StepOne™ Real-Time PCR System (Applied Biosystems, Carlsbad, CA, USA). qPCR primers: Human fascin, 5′-aaaagtgtgccttccgtacc-3′ and 5′-cccattcttcttggaggtca-3′; GAPDH, 5′-atcaagaaggtggtgaagca-3′ and 5′-gtcgctgttgaagtcagagga-3′.

### Western blotting

Cell protein concentrations were quantitated using a Bio-Rad assay (Bio-Rad, Hercules, CA, USA). Proteins were resolved by 10% SDS-PAGE and transferred to a polyvinylidene fluoride membrane (Millipore, Billerica, MA, USA). The membrane was probed sequentially with the antibodies. Anti-fascin, anti-activated-Notch1, anti-activated-Notch2, anti-Hes-1, anti-STAT3, anti-pSTAT3, anti-MMP-2 and -9, anti-NF-κB p50 and anti-GAPDH antibodies were used. Blots were developed using chemiluminescence with the LAS-4000 Imaging system (Fujifilm, Tokyo, Japan).

### RNA interference (RNAi)

RNAi was performed using small interfering RNA (siRNA) against target genes, as described previously (16–18). RNAi was performed using human STAT3 siRNA oligonucleotides from Qiagen (Hilden, Germany) and a negative control siRNA. NF-κB siRNA was from Qiagen and cells were transfected using HiPerFect transfection reagent (Qiagen).

### In vitro scratch wound healing assay

Cells were allowed to grow to confluence and cultured in serum-free medium for 12 h, prior to scratching with a sterile pipette tip. Cells were washed twice with growth medium to remove cell debris. Next, the culture medium was replaced with growth medium with 5% FBS to minimize cell proliferation. Wound areas were photographed and analyzed using the IPP 6.0 system (Intel, Santa Clara, CA, USA) at a magnification of ×100.

### Cell migration and invasion assays

Cell migration was assessed using Transwell Permeable Supports (Corning Inc., Corning, NY, USA). Briefly, cells were allowed to grow to confluence. In total, 5×10^4^ cells/well were resuspended in 100 μl serum-free medium and plated onto uncoated 8-μm transwell filter inserts of 24-well plates in triplicate. The lower chambers contained 600 μl medium containing 15% FBS as a chemoattractant. Nonmigratory cells in the upper chamber were removed with a cotton swab following incubation for 16 h. Cells on the bottom side were fixed in 100% methanol and stained with 0.5 μg/ml DAPI for 5 min. Cells were counted using a fluorescence microscope (Nikon Eclipse 80i; Nikon, Tokyo, Japan) in five random fields. For evaluation of cell invasion, cells were allowed to invade Matrigel-coated transwell filters. At the end of the experiments, invaded cells on the bottom of the membrane were incubated with 0.1% crystal violet solution and dissolved in 20% acetic acid. Finally, 100 μl dye mixture was transferred to a 96-well plate for absorbance readings at 560 nm.

### GC xenograft model

All animal experiments were approved by the Institutional Animal Care and Use Committee of Taizhou University (Taizhou, China) and the study was approved by Taizhou University Ethics Committee. MKN45 cells (5×10^6^ cells) were injected subcutaneously into the flanks of 4-week-old female athymic nude mice (Medical School Laboratory Animal Center, Zhejiang University, Zhejiang, China). Tumors became palpable (at ~75 mm^3^) within a week following injection of tumor cells and animals were randomly assigned to various treatment groups (n=7 in each group). Nude mice were injected intraperitoneally with AG490 alone (20 mg/kg). For single-agent treatment, a vehicle was administered in place of AG490 with the same schedule. Tumor size was calculated using the following formula: (width^2^ × length)/2. At the end of the experiment, tumors were resected from mice and the presence of liver metastasis was determined by hematoxylin-eosin (HE) staining.

### Statistical analysis

All data were presented as the mean ± standard deviation. Statistical significance was determined by Student’s t-test for paired or unpaired data as appropriate. P<0.05 was considered to indicate a statistically significant difference. All analyses were performed using SPSS 16.0 (SPSS Inc., Chicago, IL, USA).

## Results

### Fascin is directly regulated by STAT3 in response to IL-6 in MKN45 cells

**Figure 1:**
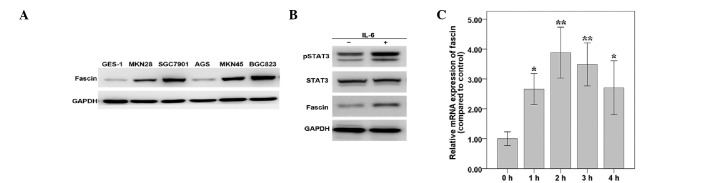
Fascin and STAT3 are upregulated by IL-6 in MKN45 cells. (A) Fascin expression of five GC cell lines and the GES-1 cell line was determined by western blotting. (B) MKN45 cells were treated with IL-6 for 1 h and the expression levels of fascin, total STAT3 and pSTAT3 (Tyr705) were detected using western blotting. (C) Quantitative polymerase chain reaction analysis measured fascin mRNA levels in MKN45 cells treated with IL-6 for 1, 2, 3 or 4 h. Results were standardized to GAPDH and shown relative to the untreated sample. Results represent three independent experiments performed in triplicate. STAT3, signal transducer and activator of transcription 3; pSTAT3, phosphotyrosine STAT3; IL-6, interleukin-6; GC, gastric carcinoma. ^*^P<0.05, ^**^P<0.01 vs. control.

**Figure 2:**
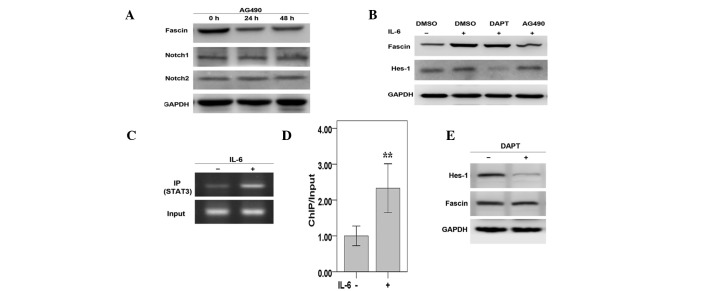
Fascin is directly regulated by STAT3 in response to IL-6 in MKN45 cells. (A) Western blotting was performed to assess protein levels of fascin, Notch1 and Notch2 in MKN45 cells treated with 50 μM AG490 for 24–48 h. (B) Cells were incubated with IL-6 and/or AG490/DAPT and western blotting was performed. (C) Cells were incubated with IL-6 and ChIP assays were performed with STAT3 antibody and primers flanking a potential STAT3 site in the human fascin promoter. (D) Results from (C) were quantitated by densitometry and expressed as ChIP/input with the untreated sample. (E) The Hes-1 and fascin expression levels in MKN45 cells treated with DAPT were assessed by western blotting. Results represent three independent experiments performed in triplicate. ^**^P<0.01, vs. control. DMSO, dimethyl sulfoxide; IL-6, interleukin-6; Hes-1, hairy and enhancer of split-1; STAT3, signal transducer and activator of transcription 3; ChIP, chromatin immunoprecipitation.

Fascin expression was determined in the GC cell lines, MKN28, SGC7901, AGS, MKN45 and BGC823, and also in the immortalized human gastric mucosal epithelial cell line, GES-1. Fascin was highly expressed in the majority of GC cell lines (Fig. 1A). Next, the relationship between fascin expression and STAT3 activation in MKN45 cells was analyzed, using IL-6 as a stimulating factor. Results showed that IL-6 induced protein expression of tyrosine phosphorylated STAT3 and fascin, with no effect on total STAT3 protein levels (Fig. 1B). However, as with the typical pattern of STAT3-target genes, fascin mRNA expression increased rapidly following IL-6 treatment and peaked at ~3-fold, after 2 h of treatment, but later decreased (Fig. 1C). Next, the AG490 JAK2/STAT3 inhibitor was used to inhibit STAT3 activation. Results demonstrated that AG490 suppressed basal and IL-6-induced fascin expression (Fig. 2A and B). Furthermore, ChIP assays revealed that STAT3 directly bound to the fascin promoter. This binding increased ~2.5-fold when treated with IL-6 (Fig. 2C and D). Collectively, these results suggest that STAT3 regulates fascin transcription through direct binding to the fascin promoter in MKN45 cells.

### Notch signaling is not involved in fascin upregulation in MKN45 cells

To investigate whether the endogenous Notch signaling pathways are involved in promotion of fascin expression, the DAPT Notch inhibitor was used. Results showed that DAPT significantly inhibited expression of target gene Hes-1 but had no significant effect on IL-6-induced fascin expression (Fig. 2B). Basal expression of fascin was also not altered by DAPT (Fig. 2E). Furthermore, AG490 had no significant effect on the constitutive expression of Notch1 and Notch2 (Fig. 2A). These results suggest that Notch signaling pathways may not be involved in the regulation of fascin expression.

### STAT3 activation induced by IL-6 is required for fascin expression and cell migration and invasion in MKN45 cells

**Figure 3:**
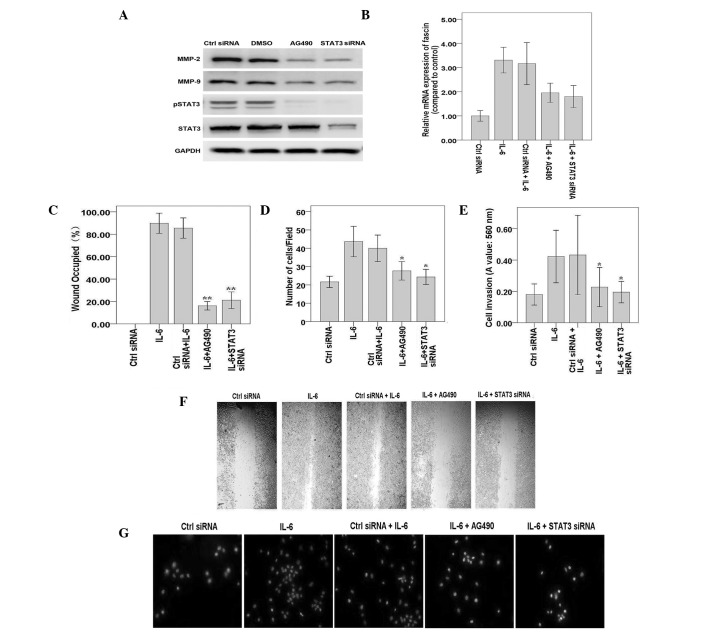
STAT3 is required for IL-6-induced expression of fascin and cell migration in MKN45 cells. (A) MKN45 cells were transfected with STAT3 siRNA or treated with AG490 and cultured for 24 h. Whole cell extracts were subjected to western blotting with antibodies against pSTAT3, total STAT3, MMP-2 and -9 and GAPDH. MKN45 cells were treated with IL-6 for 30 min. (B) MKN45 cells were transfected with STAT3 siRNA or treated with AG490 and cultured for 3 days prior to treatment with IL-6 for 30 min. mRNA levels of fascin were detected by quantitative polymerase chain reaction. Results were standardized to GAPDH and expressed as fold induction of IL-6-treated cells from three independent experiments. (C) MKN45 cells were treated as described and wound healing assays were performed. The percentage of the wound occupied in three independent experiments was calculated. (D) MKN45 cells were treated as described and cell migration assays were performed. The mean number of migrated cells in at least 5 visual fields of 3 independent experiments was calculated. (E) MKN45 cells were treated as described and cell invasion assays were performed. Invaded cells were stained and eluted for absorbance readings at 560 nm. (F) Representative experiments of wound healing assays are shown (magnification, ×100). (G) Representative experiments of cell migration assays are shown (magnification, ×100). ^*^P<0.05 and ^**^P<0.01, vs. control. MMP, matrix metalloproteinase; STAT3, signal transducer and activator of transcription 3; pSTAT3, phosphotyrosine STAT3; DMSO, dimethyl sulfoxide; IL-6, interleukin-6; siRNA, small interfering RNA.

To further investigate whether STAT3 is required for IL-6-induced fascin expression, STAT3 siRNA and AG490 were used. Western blotting showed that STAT3 expression was significantly downregulated by STAT3 siRNA and AG490 (Fig. 3A). qPCR demonstrated that IL-6-induced elevated fascin mRNA levels were significantly reversed by STAT3 siRNA transfection or AG490 treatment (Fig. 3B). These results confirm that STAT3 is required for fascin expression in MKN45 cells, in response to IL-6. In addition, results of cell migration and invasion assays showed that, compared with cells treated with IL-6 alone, cells subjected to STAT3 siRNA transfection or AG490 incubation migrated and invaded less efficiently (Fig. 3C–E). Representative results are shown in Fig. 3F and G. Consistent with this, expression of MMP-2 and -9 significantly decreased when treated with AG490 and STAT3 siRNA (Fig. 3A).

### STAT3 is required for NF-κB recruitment to the fascin promoter in response to IL-6 in MKN45 cells

**Figure 4:**
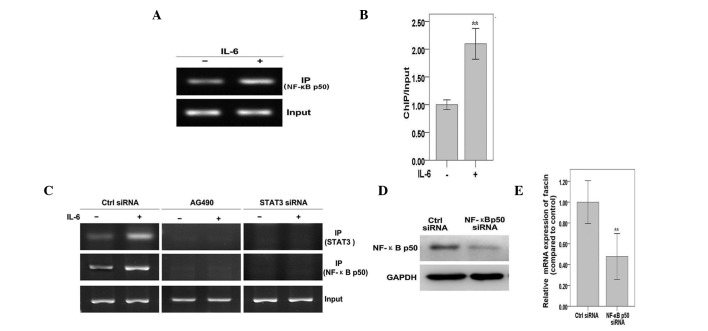
NF-κB binds to the fascin promoter in response to IL-6 in a STAT3-dependent manner. (A) MKN45 cells were treated with IL-6 for 30 min and ChIP assays were performed with an NF-κB antibody and primers flanking the potential NF-κB binding site. (B) Results from (A) were quantitated by densitometry and expressed as ChIP/input with the untreated sample. (C) MKN45 cells were transfected with STAT3 siRNA or AG490 and cultured for 3 days. Cells were treated with IL-6 for 30 min and ChIP assays were performed with STAT3 or NF-κB antibodies and primers flanking the potential STAT3/NF-κB binding sites. (D) MKN45 cells were transfected with NF-κB p50 siRNA and cultured for 4 days. Whole cell extracts were prepared and western blotting was performed with antibodies against NF-κB p50 and GAPDH. (E) MKN45 cells transfected with control or NF-κB p50 siRNA were treated with IL-6 for 30 min. RNA was subjected to quantitative polymerase chain reaction for fascin and GAPDH. Results were standardized to GAPDH and expressed as fold induction with control siRNA. Values represent the mean ± standard deviation. ^**^P<0.01, vs. control. IL-6, interleukin-6; NF-κB, nuclear factor-κB; ChIP, chromatin immunoprecipitation; siRNA, small interfering RNA; STAT3, signal transducer and activator of transcription 3.

To investigate the possible cross-talk mechanisms between the STAT3 and NF-κB pathways, in response to IL-6 treatment in MKN45 cells, ChIP assays were performed. A significant increase in NF-κB p50 bound to the fascin promoter was found when cells were treated with IL-6 (Fig. 4A and B). However, in STAT3 siRNA-transfected cells and AG490-treated cells, there was weak or no STAT3 and NF-κB p50 binding to the fascin promoter in response to IL-6 treatment (Fig. 4C). This indicates that NF-κB p50 binds to the fascin promoter in a STAT3-dependent manner. In addition, NF-κB p50 silencing resulted in reduced fascin mRNA expression (Fig. 4D and E).

### STAT3 inhibition suppresses in vivo growth and metastasis of GC cells

**Figure 5:**
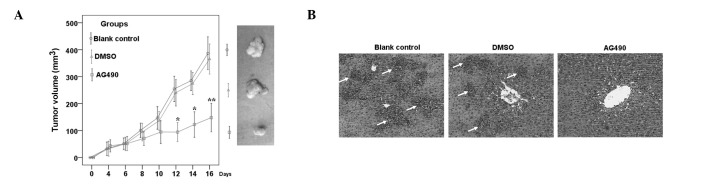
Therapeutic effects of STAT3 inhibition by AG490 on the growth and metastasis of tumors. (A) Established MKN45 tumors were measured every 2 days. Nude mice were injected intraperitoneally with AG490 (20 mg/kg), DMSO or left untreated as a control. Representative tumors were resected from mice and photographed. (B) Representative hematoxylin and eosin staining of liver metastasis (arrows) at 16 days in MKN45 xenograft model (magnification, ×200). DMSO, dimethyl sulfoxide; STAT3, signal transducer and activator of transcription 3.

To determine the role of STAT3 in tumor growth and metastasis in animals, MKN45-xenografts were established in BALB/c-nu mice. It was found that STAT3 inhibition by AG490 significantly inhibited tumor growth, compared with DMSO-treated or untreated controls (Fig. 5A). HE staining of liver tissue revealed that STAT3 inhibition caused a reduction in liver metastasis [28.57% (2/7)] compared with the control group [85.71% (6/7)] (Fig. 5B).

## Discussion

Previous data have suggested that fascin expression is abnormally high in a number of metastatic cancers, and commonly correlates with the aggressive behavior of tumor cells (6,19). In human GC, fascin expression significantly correlates with serosal invasion, histopathological grading, lymph node metastasis, tumor-node-metastasis stage and recurrence (8,20). However, to the best of our knowledge, the present study is the first to investigate the underlying cellular and molecular mechanisms of fascin expression in GC. Local invasion and lymphatic metastasis are frequent events in human GC and are associated with various cytokines, including IL-6 (21). JAK2/STAT3 is one of the major signaling pathways triggered by IL-6 and is constitutively activated in numerous cancer cell lines and types, including GC tissues (22). Aberrantly activated STAT3 has been found to enhance metastasis of tumors (23). In the present study, the hypothesis that fascin may be an important downstream effector of IL-6-regulated signaling, and that IL-6/STAT3 may sustain the basic level of fascin and upregulate fascin expression in MKN45 cells, was put forward.

For metastatic tumor cells, in addition to enhancement of tumor cell motility (24), the ability to penetrate the extracellular matrix (ECM) is crucial (25). Proteins which are secreted by tumor cells, for example MMP-2 and -9, can destroy the ECM and facilitate tumor cell invasion and metastasis (25). Al-Alwan *et al* showed that MMP-2 and -9 can be upregulated by fascin (26). Fascin and STAT3 can activate metastasis-associated molecules, including MMPs (26,27), but the exact mechanisms by which IL-6-induced STAT3 activation and fascin expression lead to cell migration and invasion are unclear. In order to elucidate this, the JAK2/STAT3 signaling pathway was downregulated in the present study, as IL-6 and STAT3 work together in the tumor microenvironment to promote several cancer hallmarks, for example increased proliferation, survival and invasion (28). The levels of MMP-2 and -9, which are critical for the execution of invasion and metastasis (29), were significantly decreased by STAT3 inhibition. STAT3 inhibition suppressed *in vivo* liver metastasis of MKN45 cells, suggesting that STAT3 may be indispensable for GC cell invasion and metastasis.

The Notch/Hes pathway crosstalk with STAT3 is also implicated in gastric carcinogenesis (30) and signaling has been reported as a reciprocal regulatory loop in the control of GC metastasis (31). As activation of Notch signaling can promote GC progression by enhancing STAT3 phosphorylation (31), we hypothesized that the Notch/Hes pathway may also be involved in the effects of IL-6 and the promotion of fascin expression in MNK45 cells. Notably, expression levels of the Notch target gene, Hes-1*,* were significantly suppressed by Notch inhibitor DAPT, but the downregulation of Notch/Hes signaling did not alter basal or IL-6-induced fascin expression (Fig. 2B and E). Furthermore, blockade of JAK/STAT3 with AG490 had no significant effect on the constitutive expression of Notch1 and Notch2 (Fig. 2A) and IL-6-induced expression of Hes-1 (Fig. 2B). These results suggest that the Notch/Hes signaling pathway may not be involved in the effects of IL-6 and is not associated with STAT3 or fascin in MNK45 cells.

The NF-κB transcription factor is able to recruit unphosphorylated STAT3 to promoters to activate transcription (32). Additionally, the NF-κB pathway positively regulates the expression of fascin (33) and can enhance metastasis of numerous tumor types (34). However, whether NF-κB has a direct effect on regulation of the STAT3-fascin loop of GC remains unclear. Therefore, further experiments were performed and data showed that NF-κB is recruited to the fascin promoter of GC cells in a STAT3-dependent manner in response to IL-6 treatment. These results suggest that, similar to STAT3, NF-κB is required for IL-6-induced expression of fascin and functions at the STAT3-dependent enhancer to increase fascin expression and promote GC metastasis. However, the exact role of NF-κB in fascin transcription in GC has not yet been established. Studies of STAT3-regulated expression of fascin will provide new insight into the mechanisms by which IL-6 promotes GC metastasis, in which multiple factors contribute to the critical step of primary tumor metastasis.

Whilst STAT3 and NF-κB are two parallel signaling pathways in human cells, the present study found that they were related and involved in the regulation of human GC metastasis. In addressing the cross-talk mechanisms between these two signaling pathways, possible links and fascin promoter activity were also analyzed. Further detailed analyses of the fascin promoter and specific interactions between transcription factors, for example STAT3 and NF-κB, may also identify potential drug targets to block metastasis. It has previously been shown that STAT3 regulates Mucin-4 expression to promote GC metastasis (11). Thus, it is possible that, in the presence of fascin, STAT3 may regulate other genes that function in GC metastasis.

The present study has shown that STAT3 may act by positively regulating fascin expression, NF-κB activity and subsequent augmentation of cell migration and invasion. By contrast, other unknown pathways may also be involved in regulating fascin transcriptional activity via NF-κB. The present study demonstrates a clear role for STAT3 in regulating GC metastasis, partially through modification of the expression of metastasis-associated genes, therefore making fascin a good target for therapeutic intervention in metastatic GC cells. Improved understanding of the fascin gene and the impinging signaling cascades is required to improve understanding of STAT3-driven processes contributing to increased fascin levels and, consequently, to more aggressive cellular behavior of GCs.
